# CoO–Co Heterojunction Covered with Carbon Enables Highly Efficient Integration of Hydrogen Evolution and 5-Hydroxymethylfurfural Oxidation

**DOI:** 10.3390/molecules28073040

**Published:** 2023-03-29

**Authors:** Lei Zhao, Shichao Du, Rui Gong, Wanqi Jia, Zhimin Chen, Zhiyu Ren

**Affiliations:** Key Laboratory of Functional Inorganic Material Chemistry (Ministry of Education of China), School of Chemistry and Materials Science, Heilongjiang University, Harbin 150080, China

**Keywords:** integrated electrolysis, heterojunction, electrochemical hydrogen evolution, biomass electrooxidation

## Abstract

The renewable-energy-driven integration of hydrogen production and biomass conversion into value-added products is desirable for the current global energy transition, but still a challenge. Herein, carbon-coated CoO–Co heterojunction arrays were built on copper foam (CoO–Co@C/CF) by the carbothermal reduction to catalyze the hydrogen evolution reaction (HER) coupled with a 5-hydroxymethylfurfural electrooxidation reaction (HMFEOR). The electronic modulation induced by the CoO–Co heterojunction endows CoO–Co@C/CF with a powerful catalytic ability. CoO–Co@C/CF is energetic for HER, yielding an overpotential of 69 mV at 10 mA·cm^−1^ and Tafel slope of 58 mV·dec^−1^. Meanwhile, CoO–Co@C/CF delivers an excellent electrochemical activity for the selective conversion from HMF into 2,5-furandicarboxylic acid (FDCA), achieving a conversion of 100%, FDCA yield of 99.4% and faradaic efficiency of 99.4% at the lower oxidation potential, along with an excellent cycling stability. The integrated CoO–Co@C/CF||CoO–Co@C/CF configuration actualizes the H_2_O–HMF-coupled electrolysis at a satisfactory cell voltage of 1.448 V at 10 mA·cm^−2^. This work highlights the feasibility of engineering double active sites for the coupled electrolytic system.

## 1. Introduction

Renewable-energy-driven electrochemical water splitting for hydrogen production is a promising avenue for synchronously weakening our dependence on conventional fossil fuels and mitigating the associated environmental issues by providing affordable clean energy [1,2,3,4]. For electrolytic water splitting, relative of hydrogen evolution reaction (HER), oxygen evolution reaction (OER) at the anode generally accounts for most of the energy consumption of the system, by virtue of its high-barrier thermodynamics and the sluggish reaction kinetics [3,5,6,7,8,9]. Moreover, the low-value product, O_2_, limits the economy considerably [10,11,12]. Therefore, it is necessary to replace the OER with the other anode reactions that occur at a lower applied potential and produce high-value products, thus meeting the requirements of low energy consumption and high economic efficiency.

Five-hydroxymethylfurfural (HMF) is a common renewable biomass that is produced by the dehydration of carbohydrates such as glucose, fructose, and cellulose [13,14,15,16,17,18]. Owing to the advantages of bio-based chemicals and structures such as terephthalic acid, the typical oxidation product of HMF, 2,5-furandicarboxylic acid (FDCA) has become a research hotspot in polyester industry in recent years [14,19,20,21]. In industry, the conversion from HMF to FDCA is an energy-intensive process, which should be carried out at high temperatures (30–130 °C) and high pressures of oxygen (e.g., 0.3–2.0 MPa) using precious metal-based catalysts (e.g., Au, Pt, Ru, and Pd) [16,22,23]. On the contrary, the electrical energy enables HMF to be oxidized without the harsh reaction conditions. It is gratifying that, as the anodic substitution reaction, the HMF electrooxidation reaction (HMFEOR) can be properly implemented under a lower applied potential, which benefits from its priority in thermodynamics and kinetics [18,24,25]. Therefore, it is foreseeable that integrating HER and HMFEOR in the same electrolyzer can reduce the energy consumption of the reaction system [19,26,27]. More importantly, such an integrated electrolytic system can produce H_2_ and high value-added chemicals at the same time, thus maximizing economic efficiency [13,28,29].

Developing the suitable bifunctional catalysts, which should contain two different active sites to drive HER and HMFEOR at the cathode and anode, is the necessary to realize integrated electrolysis [14,30,31,32]. In view of the scarcity and the high price of precious metals, transition metal-based electrocatalysts with a fascinating *d*-band electronic structure have attracted wide attention recently [32,33,34,35]. In particular, the multi-valence feature of transition metals provides the possibility to construct multiple different catalytic sites in the same catalyst [21,32,36,37]. Previous studies have corroborated that, due to the carbothermal reduction reaction, some transition-metal species can be reduced by the carbon generated from the peripheral organic ligands at a high temperature, thus producing the heterojunction of metal and metal oxide [38,39,40]. The generated metal and metal oxide can be used as catalytic sites to drive the cathode and anode reactions, respectively [41,42]. Meanwhile, the metal–metal oxide heterojunction can alter the electronic distribution of the active sites, resulting in an inspiring electrocatalytic activity [31,43,44,45].

In view of the above considerations, the CoO–Co heterojunction arrays covered with carbon were designed and directly constructed on copper foam (CoO–Co@C/CF), which efficiently achieves the integration of HER and HMFEOR in an alkaline system. In the anode, CoO–Co@C/CF can catalyze HMFEOR to produce FDCA with a satisfying yield and a Faraday efficiency (FE) (>99%) at a low applied potential of 1.34 V vs. the reversible hydrogen electrode (RHE). In the cathode, the HER overpotential at 10 mA·cm^−2^ occurring on CoO–Co@C/CF is about 69 mV, accompanying a small Tafel slope of 58 mV·dec^−1^. Specifically, the electrolyzer assembled with CoO–Co@C/CF for the coupled H_2_ and FDCA production only requires a voltage of 1.448 V at 10 mA·cm^−2^, lower than that for water splitting (1.655 V at 10 mA·cm^−2^). We expect that the bifunctional two-electrode electrolyzer can couple HER with many other organic reactions for establishing a valuable electrochemical reforming system.

## 2. Results and Discussion

### 2.1. Synthesis and Structural Characterizations of CoO–Co@C/CF

**Figure 1:**
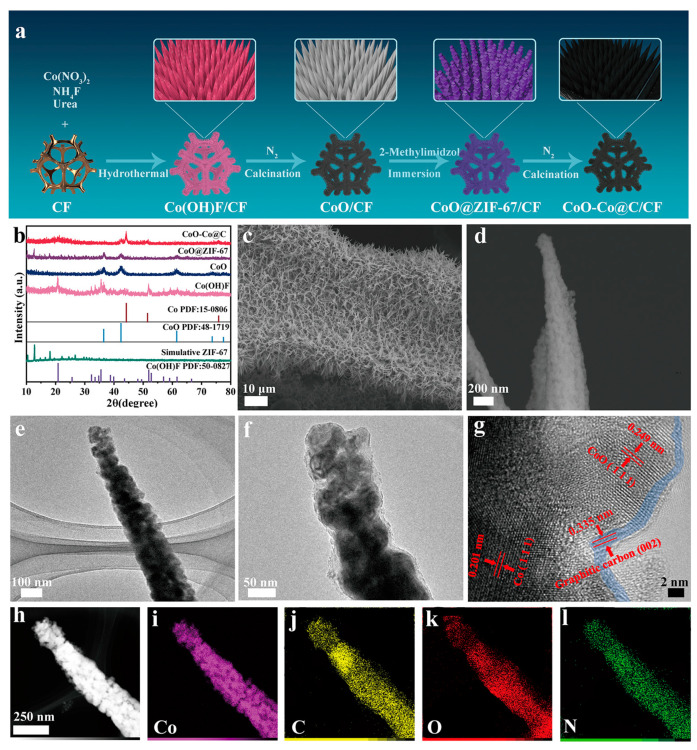
Schematic illustration of the synthetic procedure for CoO–Co@C/CF (**a**), XRD patterns of Co(OH)F, CoO, CoO@ZIF-67, and CoO–Co@C exfoliated from CF (**b**); SEM images (**c**,**d**), TEM images (**e**,**f**) and high-resolution TEM image (**g**), TEM-EDS element mapping images (**h**–**l**) of CoO–Co@C exfoliated from CF.

CoO–Co@C/CF was prepared through a successive four-step process, as depicted in Figure 1a. The evolution of the structure and morphology were explored in detail by the X-ray diffraction (XRD) patterns, scanning electron microscopy (SEM) images and the transmission electron microscopy (TEM) images. Firstly, the uniform Co(OH)F (JCPDS no. 50-0827) cone-shaped arrays grow on the Cu foam (CF) during the hydrothermal synthesis (Figure 1b and Figure S1). After calcination in N_2_, Co(OH)F translates into CoO (JCPDS no. 48-1719) without the morphology change (Figure S2). Compared with Co(OH)F/CF, CoO/CF is a more desirable cobalt source to realize the uniform coverage of polygonal ZIF-67 on the surface, through the coordination erosion (Figures S3 and S4a–c). The TEM images reveal the formation of a core–shell structure in which the CoO core is tightly wrapped inside the ZIF-67 shell (Figure S4d–f). Finally, during the carbothermal reduction in an inert atmosphere, CoO@ZIF-67 further converts into carbon-coated CoO–Co heterojunction on the surface of CF (CoO–Co@C/CF). In the XRD pattern of CoO–Co@C/CF (Figure 1b), the diffraction peaks at 36.5°, 42.4°, and 61.5° are well coincident with (111), (200), and (220) planes of CoO (JCPDS no. 48-1719), while the peaks located at 2θ = 44.2°, 51.5°, and 75.9° are indexed to (111), (200), and (220) crystal facets of metallic Co (JCPDS no. 15-0806), indicating the coexistence of the two phases. There are clear peaks at 1336.7, 1587.9, and 2880.5 cm^−1^ assigned to the D band, G band, and 2D band of graphitic carbon in the Raman spectrum of CoO–Co@C/CF (Figure S5), which is different from that of CoO@ZIF-67/CF [27]. The 2D peak widens and the intensity ratio of the D and G bands (I_D_/I_G_) is about 0.89, indicating that the carbon located near to the Co species is graphitized to a certain degree during the calcination in N_2_. The graphitic carbon layer in CoO–Co@C/CF is not only beneficial to improving the electrical conductivity, but also protects the catalytic sites during reactions. As shown in SEM and TEM images (Figure 1c–f), the obtained CoO–Co@C/CF presents the uniform nano cones assembled with particles. The obvious lattice fringes of 0.201 and 0.249 nm corresponding to Co (111) and CoO (111) can be observed in the high-resolution TEM images (Figure 1g). Additionally, the lattice fringes with interplanar spacing of 0.335, which can be assigned to the graphitic carbon (002), emerge in the outermost layer (about 2 nm). It also confirms the formation of graphitized carbon, well matching the Raman result. The TEM-energy-dispersive X-ray spectroscopy (STEM-EDX) mapping images (Figure 1h–k) suggest that Co, C, and O elements are all uniformly distributed on the surface of the whole nano cones. For comparison, Co@C/CF and CoO@C/CF were also prepared by changing the calcination atmosphere into H_2_ or air, respectively (see the Materials and Chemicals section for details). In the XRD patterns (Figure S6), the diffraction peaks of Co@C and CoO@C are well indexed to metallic Co (JCPDS no. 15–0806) and CoO (JCPDS no. 48–1719), respectively. Meanwhile, there is no obvious difference in the morphology of Co@C/CF, CoO@C/CF, and CoO–Co@C/CF (Figure S7).

**Figure 2:**
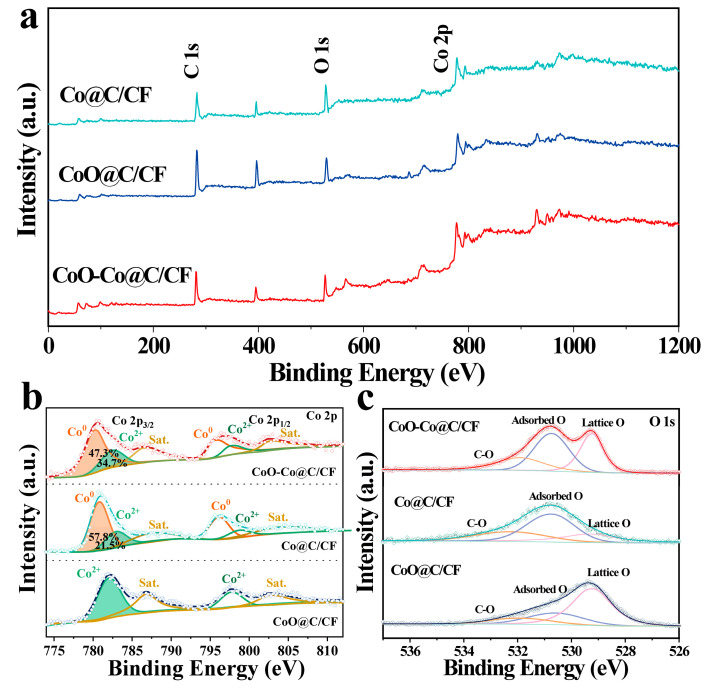
XPS survey spectra (**a**), Co 2p spectra (**b**) and O 1s spectra (**c**) of CoO–Co@C/CF, Co@C/CF, and CoO@C/CF.

The elemental composition and valence states of CoO–Co@C/CF, Co@C/CF, and CoO@C/CF were analyzed by the X-ray photoelectron spectroscopy (XPS). Figure 2a compares the XPS survey spectra of CoO–Co@C/CF, Co@C/CF, and CoO@C/CF, which shows the existence of C, O, and Co elements in all samples. In the Co 2p spectrum of CoO–Co@C/CF (Figure 2b), the peaks at 780.2 eV and 795.9 eV can be assigned to metallic Co, the peaks at 782.6 eV and 798.1 eV belong to Co^2+^, and the peaks at 786.7 eV and 802.7 eV are satellites [25,46,47]. It is worth noting that, compared with metallic Co in Co@C/CF, a slight shift to the low binding energy occurs in the Co 2p XPS of CoO–Co@C/CF, while the binding energy of Co^2+^ in CoO–Co@C/CF slightly moves in a positive direction relative to that in CoO@C/CF, which signifies the strong electron interaction between metallic Co and CoO. The content of metallic Co decreases in the order of Co@C/CF > CoO–Co@C/CF > CoO@C/CF, which is exactly the opposite of the Co^2+^. Figure 2c compares the O 1s XPS of the three studied samples. The peaks at 529.2, 531.3, and 531.92 eV belong to the lattice oxygen, adsorbed oxygen, and carbon oxygen, respectively. Moreover, the content of lattice oxygen decreases with the increase of metal cobalt content. The results from XRD and XPS indicate the coexistence of CoO and Co in the CoO–Co@C/CF.

### 2.2. Electrochemical Activity of CoO–Co@C/CF towards HMF Oxidation

**Figure 3:**
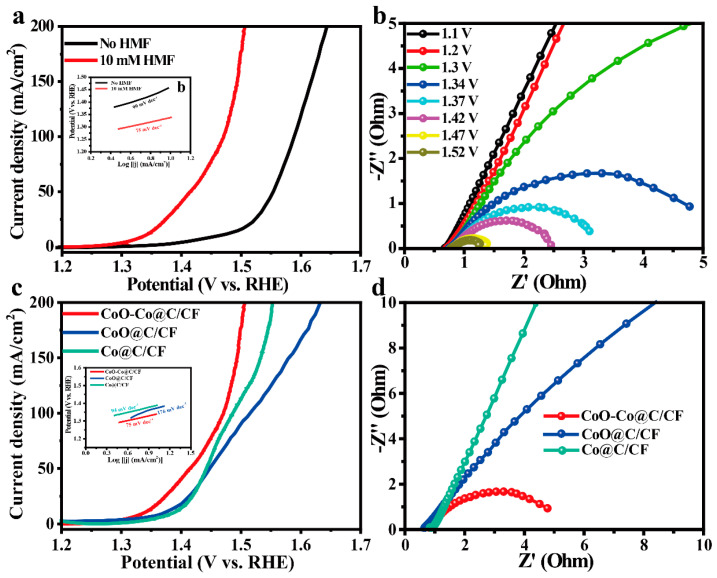
(**a**) iR-compensated polarization curves of HMFEOR over CoO–Co@C/CF in 1.0 M KOH with/without 10 mM HMF at the scan rate of 5 mV·s^−1^ (inset: corresponding Tafel plots). (**b**) Potential-dependent Nyquist plots of CoO–Co@C/CF in 1.0 M KOH with 10 mM HMF. (**c**) Polarization curves of HMFEOR over CoO–Co@C/CF, CoO@C/CF, and Co@C/CF in 1.0 M KOH with 10 mM HMF at the scan rate of 5 mV·s^−1^ (inset: corresponding Tafel plots). (**d**) Nyquist plots at 1.34 V of CoO–Co@C/CF CoO@C/CF, and Co@C/CF in 1.0 M KOH with 10 mM HMF.

The electrocatalytic ability of CoO–Co@C/CF towards HMFEOR and the major competing reaction, OER, were measured by typical three-electrode system in an H-type cell separated by an anion exchange membrane (AEM). Figure 3a compares the electrocatalytic activities of CoO–Co@C/CF towards HMFEOR and OER. When HMF is added into the reaction system, the onset potential decreases sharply from 1.35 V vs. RHE to 1.25 V vs. RHE. Only an applied potentials of 1.34 V are required for CoO–Co@C/CF to yield the HMFEOR current density of 10 mA·cm^−2^, lower than that required in OER (1.46 V vs. RHE). Moreover, with the concentrations of HMF increasing, the superiority of CoO–Co@C/CF in catalyzing HMFEOR is more significant (Figure S8). As shown in Figure S9, the catalytic activity towards HMFEOR gradually improved along with the structure evolution. It follows that CoO–Co@C/CF is more sensitive to HMFEOR than OER. The corresponding Tafel slope of HMFEOR on CoO–Co@C/CF is 75 mV·dec^−1^, lower than that of OER (99 mV·dec^−1^), which suggests that CoO–Co@C/CF has more rapid reaction kinetics in HMFEOR (the insert in Figure 3a). The intrinsic kinetics of CoO–Co@C/CF was further explored by the potential-dependent electrochemical impedance spectroscopy (EIS), measured in 1.0 M KOH with 10 mM HMF (Figure 3b). The equivalent circuit model of Nyquist plots is depicted in Figure S9. At the low applied potentials (from 1.10 to 1.20 V vs. RHE), the Nyquist plots show approximated vertical lines, indicating the high charge-transfer resistance (R_ct_). When the applied potential increases to 1.3 V vs. RHE, an incomplete arc appears in the Nyquist plots, signifying the beginning of the Faradaic reaction. Increasing the applied potential to 1.34–1.52 V vs. RHE, the semi-arc gradually completes and the radius of the arc became smaller and smaller, indicating that the R_ct_ value decreases rapidly at lower potentials, which causes a more intensive HMFEOR reaction. Compared with Co@C/CF and CoO@C/CF, CoO–Co@C/CF can arouse HMFEOR at a lower potential and deliver a high current density of HMFEOR at each applied potential, highlighting the superiority of CoO–Co@C/CF towards HMFEOR in the thermodynamics (Figure 3c). The Tafel slope of HMFEOR on CoO–Co@C/CF is lower than that on Co@C/CF and CoO@C/CF, suggesting rapid reaction kinetics in HMFEOR (the insert in Figure 3c). Meanwhile, the R_ct_ values of CoO–Co@C/CF are the smallest among all of the catalysts, corroborating that the CoO–Co heterojunction structure and graphitic carbon effectively expedite the charge transfer between CoO–Co@C/CF and reactants/intermediates in HMFEOR (Figure 3d and Table S1).

**Figure 4:**
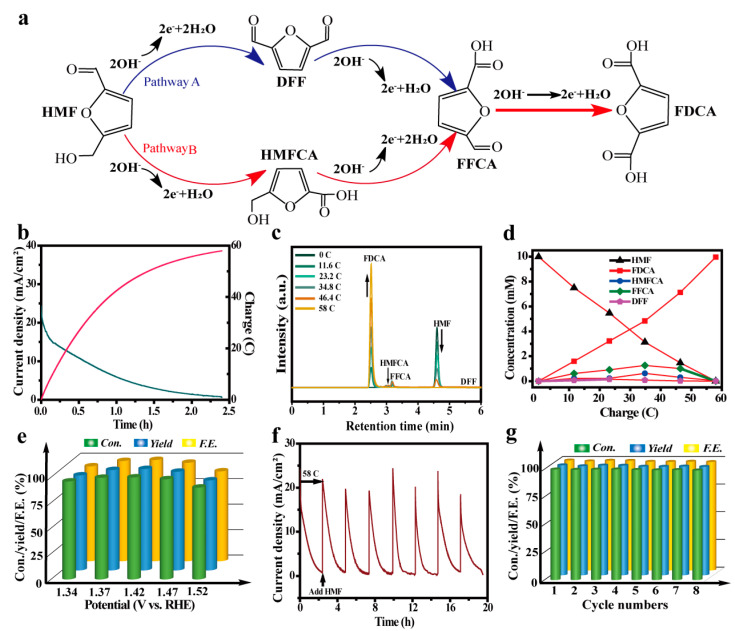
(**a**) Two possible pathways of HMFEOR to FDCA. (**b**) I−t curve for CoO–Co@C/CF at constant potential of 1.37 V vs. RHE in 1.0 M KOH with 10 mM HMF by passing the charge of 58 C. (**c**) HPLC traces of HMFEOR over CoO–Co@C/CF at 1.37 V vs. RHE in 1.0 M KOH with 10 mM HMF at various reaction times. (**d**) Concentrations of HMF, intermediates, and products during HMFEOR over CoO@C/CF. (**e**) HMF conversion, FDCA yield, and FE of HMFEOR at various potentials. (**f**) I−t curve of HMFEOR over CoO–Co@C/CF at 1.37 V vs. RHE with the intermittent addition of 10 mM HMF. (**g**) HMF conversion, FDCA yield, and FE of HMFEOR over CoO–Co@C/CF in eight successive cycles.

Generally, there are two possible pathways for HMFEOR [48,49]. In the first step, the aldehyde group of HMF is oxidized to yield 5-hydroxymethyl-2-furancarboxylic acid (HMFCA), or the hydroxyl group of HMF is oxidized to yield 2,5-diformylfuran (DFF) (Figure 4a). Subsequently, two intermediates (HMFCA and DFF) are further oxidized to 5-formyl-2-furancarboxylic acid (FFCA) and finally to FDCA. The polarization curves of CoO–Co@C/CF towards various biomass (HMF, HMFCA, DFF, FFCA, or FDCA) oxidation are depicted in Figure S10. By comparison, the catalytic ability of CoO–Co@C/CF towards HMF and intermediates (HMFCA, DFF, and FFCA) is superior to that towards FDCA. In order to identify the reaction pathway of HMFEOR, the chronoamperometry experiments were carried out at a constant potential of 1.37 V vs. RHE and the content of reactants/products was quantified by the standard curves determined by the high-performance liquid chromatography measurements (HPLC) (Figures S11–S15). Theoretically, owing to the complex six-electron transfer, a charge of 58 C is required to convert the HMF (10 mM, 10 mL) into FDCA completely, which can be implemented by applying a potential of 1.37 V vs. RHE for 2.4 h (Figure 4b). The superficial evidence from the color change of the electrolyte suggests the conversion from HMF to FDCA (Figure S16). As shown in Figure 4c, the HPLC signals of HMF and FDCA display a diametrically opposite trend with the prolonging of the reaction time, demonstrating the gradual conversion from HMF to FDCA. When the charge reaches 58 C, the HPLC signal of HMF disappears, while that of FDCA increases to the maximum. It is worth noting that there is no response of DFF in HPLC, illustrating that the implementation of HMFEOR on CoO–Co@C/CF goes through HMF → HMFCA → FFCA→ FDCA. According to the standard curves, the concentration changes of HMF, DFF, HMFCA, FFCA, and FDCA during the electrolysis are depicted in Figure 4d. CoO–Co@C/CF delivers an excellent catalytic activity with the HMF conversion of 100%, FDCA yield of 99.4%, and FE of 99.4%. Different potentials (from 1.34 V to 1.52 V) were applied to HMF conversion with the constant charge of 58 C. With the increase in applied potentials, the time required for HMF oxidation is significantly reduced (Figure 4e and Figure S17). Furthermore, when the applied potential increases to 1.52 V vs. RHE, the decline in HMF conversion, FDCA yield, and FE is obvious and some bubbles were produced on the surface of working electrode, which indicates that part of the charges have been used for water oxidation. The electrolysis cycles (at a constant potential of 1.37 V vs. RHE) are continuously operated to investigate the stability and durability of CoO–Co@C/CF. During the multiple cycle tests, a nearly constant HMF conversion (100%), FDCA yield (99.4%), and FE (99.4%) can be achieved, manifesting the potential of CoO–Co@C/CF in practical applications (Figure 4g). CoO–Co@C/CF delivers a prominent ability of catalyzing HMFEOR, especially in the selectivity and FE, which ranks among the best Co-based catalysts reported so far (Table S2).

### 2.3. Electrochemical Activity of CoO–Co@C/CF towards HER

**Figure 5:**
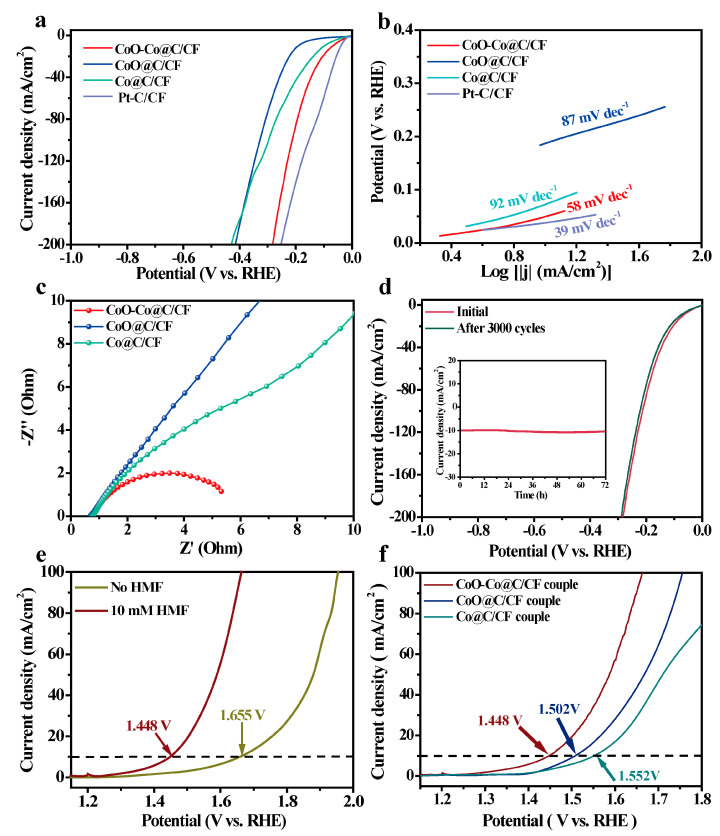
(**a**) iR-compensated polarization curves and (**b**) Tafel slopes of HER over CoO–Co@C/CF, CoO/CF, Co/CF, and Pt−C/CF in 1.0 M KOH at a scan rate of 5 mV·s^−1^. (**c**) Nyquist plots at 0.080 V vs. RHE of CoO–Co@C/CF, CoO@C/CF, Co@C/CF, and Pt−C/CF in 1.0 M KOH. (**d**) Polarization curves of CoO–Co@C/CF before and after 3000 cycles (Inset: I-t curve of CoO–Co@C/CF at *η*_10_ for 72 h). (**e**) Polarization curves of CoO–Co@C/CF||CoO–Co@C/CF systems in 1.0 M KOH with/without 10 mm HMF. (**f**) Polarization curves of CoO–Co@C/CF, CoO@C/CF, and Co@C/CF couples in 1.0 M KOH with 10 mM HMF.

The electrocatalytic HER tests of all samples were also conducted in the alkaline electrolyte. CoO–Co@C/CF displays a satisfactory HER activity with an onset potential of 19 mV and the low overpotentials (*η*) of 69 mV and 207 mV to reach 10 mA·cm^−2^ and 100 mA·cm^−2^, which is only higher than that of Pt-C/CF, but much lower than that of Co@C/CF (*η*_10_ = 100 mV and *η*_100_ = 296 mV) and CoO@C/CF (*η*_10_ = 189 mV and *η*_100_ = 327 mV) (Figure 5a and Table S3). Meanwhile, the HER performance of CoO–Co@C/CF is also better than the other pre-catalysts (Co(OH)F/CF, CoO/CF, and CoO@ZIF/CF) (Figure S18). Such catalytic activity of CoO–Co@C/CF ranks first among Co-based HER catalysts (Table S3).

The Tafel slope of CoO–Co@C/CF is 58 mV·dec^−1^, which is smaller than that of CoO@C/CF (87 mV·dec^−1^) and Co@C/CF (92 mV·dec^−1^), demonstrating that CoO–Co@C/CF has the rapid HER reaction kinetics of CoO–Co@C/CF (Figure 5b). The inherent features of carbon-coated CoO–Co heterojunctions positively contribute to such outstanding activity. The EIS tests corroborate that the R_ct_ value of CoO–Co@C/CF (5.2 Ω) is far less than that of Co@C/CF (26.4 Ω) and CoO@C/CF (38.3 Ω), which profits from the high conductivity capability of the heterojunction and graphitic carbon layer that accelerates the fast charge transfer between CoO–Co@C/CF and reactants/intermediates in the HER process (Figure 5c and Table S1). Additionally, after 3000 cycles in the potential range from 0 to −1 V vs. RHE (Figure 5d), the polarization curve of HER on CoO–Co@C/CF is basically consistent with the initial one. In addition, no evident decline in the current density can be observed during 72 h HER (inset in Figure 5d).

### 2.4. Integrated HMF Oxidation and H_2_ Evolution on CoO–Co@C/CF

In view of the advisable catalytic performance of CoO–Co@C/CF, the two-electrode electrolyzer was built by CoO–Co@C/CF as the anode and cathode to achieve HER and HMFEOR synchronously. Compared with water splitting, the voltage of HER-HMFEOR coupled electrolysis on the CoO–Co@C/CF||CoO–Co@C/CF system noticeably reduces by 207 mV at 10 mA·cm^−2^, highlighting the superiority of electrocatalytic coupling strategy in the energy-efficient operations (Figure 5e). Moreover, this system displays the good stability and durability in the multi-cycle tests (Figure S20). This is mainly attributed to the delightful structure stability of CoO–Co@C/CF in both HMFEOR and HER, as evidenced by the SEM images and XRD patterns after electrochemical processes (Figures S21 and S22). The performance of CoO–Co@C/CF for HER-HMFEOR-coupled electrolysis is much better than that of CoO@C/CF and Co@C/CF couples (Figure 5f) and even better than that of most reported bifunctional catalysts (Table S4).

## 3. Materials and Methods

### 3.1. Materials and Chemicals

NH_4_F, Co(NO_3_)_2_·6H_2_O, urea, 2-methylimidazole, and potassium hydroxide (KOH) were obtained from Tianjin Kemiou Chemical Reagent Co. Ltd. Hydrochloric acid (HCl), acetone was obtained from Tianjin Fuyu Chemical Reagent Co. Ltd. The 5-hydroxymethylfurfural (HMF), 5-formyl-2-furancarboxylic acid (FFCA), 5-hydroxymethyl-2-furancarboxylic acid (HMFCA), 2,5-furandicarboxylic acid (FDCA), 2,5-diformylfuran (DFF), ammonium formate, ethanediol, methanol, and ethyl alcohol were all purchased from Aladdin Chemical Co. Ltd. Additionally, several large pieces of copper foam (CF) were bought wholesale from Suzhou Jiashide Foam Metal Co. Ltd. All the deionized water (DI water, 18.2 MΩ) used in this experiment was obtained from purification through a splendid Millipore system. The well-cut size CF (e.g., 3 cm × 4 cm) was sonicated in acetone for 15 min to remove the oil contamination layer on the surface. Subsequently, in order to remove the appeared copper oxide on the surface, the CF was soaked in 1 M HCl ultrasound for 10 min, then rinsed with water after being pre-treated, and finally dried in a vacuum oven.

### 3.2. Preparation of the Electrodes

Synthesis of CoO–Co@C/CF: CoO–Co@C/CF was prepared through the successive four-step process. Typically, 2 mmol Co(NO_3_)_2_·6H_2_O, 12 mmol urea, and 8 mmol NH_4_F were dissolved into 40 mL DI water and stirred for 1 h to form a uniform solution. Then, the solution was transferred into a 50 mL Teflon-lined autoclave, and the CF (3.0 × 4.0 cm) submerged in the mixture with vigorous ultrasonic treatment for 15 min. The Teflon-lined autoclave was sealed, and heated in the oven at 120 °C for 8 h. After the autoclave was cooled down to room temperature, the reactor was removed from the oven, washed with water to neutralize and collect the specimen, followed by drying at 60 °C to obtain the rosy Co(OH)F line array on the CF, denoted as Co(OH)F/CF. The prepared Co(OH)F/CF sample was then heated to 400 °C with a ramp rate of 2 °C·min^−1^, and the temperature kept at 400 °C for 2 h, under a N_2_ atmosphere. After cooling to room temperature, the gray-black material was washed with DI water to remove impurities and dried to obtain a black CoO line array on the CF, named CoO/CF.

In a glass weighing bottle, 7.5 mmol 2-methylimidazole was dissolved into a 20 mL mixture of water and ethanediol, at a ratio of 1:1, and stirred for 30 min. Then, the CoO/CF (2.0 cm × 3.0 cm) sample was immersed in the above uniform solution. The weighing bottle was placed in a water bath and kept at a constant temperature of 30 °C for 12 h. The purple precipitates were taken out of the weighing bottle, and washed with DI water and ethanol for several times, then dried in the air. Finally, the violet ZIF-67 rod array was obtained on the CF (denoted as CoO@ZIF-67/CF). The resulting ZIF-67/CF sample was heated to 400 °C in a N_2_ atmosphere with a ramp rate of 2 °C·min^−1^, and maintained at 400 °C for 150 min. After naturally dropping to room temperature, the black material was washed with DI water to remove the impurity, and dried in an oven at 60 °C for 6 h. The final catalyst CoO–Co@C/CF was obtained.

Synthesis of CoO@C/CF and Co@C/CF: The CoO@C/CF and Co@C/CF were also synthesized as a comparison. On one hand, the ZIF-67/CF precursor was heated to 350 °C in a N_2_ atmosphere, and maintained for 30 min to obtain the CoO@C/CF. On the other hand, the prepared ZIF-67/CF was calcined in a hydrogen atmosphere at 400 °C for 150 min instead of a nitrogen atmosphere to obtain another comparison sample denoted Co@C/CF.

### 3.3. Characterizations

Using the Brockke D8 advanced diffractometer to carry out the X-ray diffraction (XRD) test and using a VG ESCALABMK II device equipped with Mg-Ka radiation (1253.6 eV) to perform the X-ray photon spectroscopy (XPS) analysis. A Hitachi S-4800 instrument with an accelerating voltage at 15 KV was used for scanning electron microscopy (SEM) test to obtain images of the prepared samples. The transmission electron microscopy (TEM) characterization was obtained from a JEM-2100 electron microscope (JEOL, Japan) with an acceleration of 200 kV. The micro-Raman spectrometer in an instrument model of Jobin Yvon HR 800 (λ = 457.9 nm) was used to perform the Raman measurements. The organics were quantitatively tested by high-performance liquid chromatography (HPLC, Wufeng LC-100C), which was equipped with an ultraviolet-visible (UV) detector (Set UV absorption wavelength at 265 nm and a 4.6 mm × 250 mm Shim-pack GWS 5 μm C 18 column). Gas chromatography (Aglient, 7820A) was used for the quantitative analysis of the amount of H_2_.

### 3.4. Electrochemical Measurements

The OER, HER, and HMFEOR measurements were performed on an electrochemical equipment (Princeton, NJ, USA) equipped with a three-electrode system, which has an H-shaped divided cell. An anion exchange membrane (AEM) was used to separate the anode and cathode. The as-prepared free-standing CoO–Co@C/CF samples were cut into 1.5 cm × 1.0 cm, as the working electrode. The Ag/AgCl electrode (saturated KCl filled) was used as the reference electrode, and the counter electrode was the Pt mesh. The electrochemical OER- and HER-related tests were conducted in 1.0 M KOH solution (pH = 13.9). In addition, 10 mL 1.0 M KOH (pH = 13.9) with 10 mM HMF was used as the electrolyte of HMFEOR. All reported potentials were adjusted to a reversible hydrogen electrode (RHE) following the equation:

(1) E (vs. RHE)=E (vs. Ag/AgCl)+E (Ag/AgCl)+0.059 pH

Prior to all electrochemistry measurements, the working electrode was scanned by 20 cyclic voltammograms curves (CVs) at 100 mV·s^−1^ in the electrolyte to obtain a steady state of electrocatalyst. The linear sweep voltammetry (LSV) was recorded in the electrolyte at a scan of 5 mV·s^−1^. The 90% iR compensation was employed in all the electrochemical measurements. The electrochemical impedance spectra (EIS) were tested by the AUTOLAB electrochemical workstation in different electrolytes and different potentials from 1 × 10^−2^ Hz to 1 × 10^5^ Hz. The Tafel slopes were calculated according to the Tafel equation as follows:

(2) $$\eta =b\;\times \log(\frac{j}{j0})$$

where *η* is the potential, *b* is the Tafel slope, *j* is the current density, and *j0* is the exchange current density.

To explore the stability of electrocatalysts, the long-time stability test of HER was carried out at 100 mV·s^−1^ by CVs in the potential region of 0 ~ −1.0 V (vs. RHE), and tested for 72 h at the potential required 10 mA·cm^−2^. The HMFEOR was carried out at room temperature, with the stirring at different potentials passing 58 C (for the sake of converting the given amount of HMF to FDCA, the required stoichiometric coulomb quantity is 58 C). The stability testing of HMFEOR was performed at 1.37 V, 8 cycles with the addition of HMF to measure conversion rate, yield, and Faraday efficiency. For the two-electrode electrolysis, CoO–Co@C/CF was employed as the electrocatalyst for both anodes and cathodes.

### 3.5. Product Quantification

In order to analyze the reactant conversion rate, the product yield, and the corresponding Faraday efficiency, the electrochemical oxidation of HMF on account of the three-electrode and two–electrode systems was performed with a potentiostatic method at different potentials (e.g., 1.37 V vs. RHE for the three-electrode test, and 1.56 V for two-electrode configuration), passing 58 C with stirring. During and after the reaction, 20 μL of the anode compartment solution was removed, mixed with 100 μL 0.2 M HCl solution, diluted with ultrapure water to 800 μL, then analyzed by HPLC (Wufeng LC-100C) with a 4.6 mm × 250 mm Shim-pack GWS 5 μm C 18 column. The ultraviolet-visible detector wavelength was set to 265 nm, while the methanol as a mobile phase A was mixed with mobile phase B, which was a 5 mM ammonium formate aqueous solution in a ratio of 3:7, and the flow rate was 1 mL·min^−1^; all of the separations lasted for 6 min. All the quantitative analysis of reactants, intermediates, and products was made by an external standard method. In the HMF oxidation process, the conversion (%) of HMF, product yield (%), and the Faraday efficiency (FE, %) of the product were calculated according to the following equations:

(3) HMF conversion (%)=n(HMF consumed)/n(HMF original) × 100%

(4) FDCA yield (%)=n(FDCA produced)/n(HMF original) × 100%

(5) FE (%)=(n × F × n(FDCA produced)/total charged passed × 100%

where *F* is the Faraday constant (96,500 C mol^−1^) and *n* is the number of electrons transferred for the final product FDCA formation.

## 4. Conclusions

In summary, the carbon-coated CoO–Co heterojunction arrays made up of nanoparticles were successfully built on CFs to couple the H_2_ production with HMF oxidation. Benefiting from the synergistic effect between the CoO–Co heterojunction and graphitic carbon, CoO–Co@C/CF is energetic for HER and HMFEOR in both thermodynamics and kinetics. The two-electrode system assembled by CoO–Co@C/CF features a small cell voltage of 1.448 V for the HER-HMFEOR-coupled electrolysis. The strategy developed herein afforded a facile way to fabricate double catalytic sites for electrocatalysis.

## Data Availability

The data are available on request from the corresponding authors.

## Supplementary Materials

The following supporting information can be downloaded at: https://www.mdpi.com/article/10.3390/molecules28073040/s1, Figures S1–S4: SEM images of Co(OH)F/CF, CoO/CF, Co(OH)F@ZIF-67/CF, and CoO@ZIF-67/CF; Figure S5: Raman spectra of the CoO-Co@C/CF and CoO@ZIF-67/CF; Figure S6: XRD patterns of CoO-Co@C/CF, CoO@C/CF and Co@C/CF; Figure S7: SEM images of CoO@C/CF; Figure S8: Polarization curves of HMFEOR over CoO-Co@C/CF in 1.0 M KOH with various concentrations of HMF; Figure S9: The equivalent circuit model of Nyquist plots; Figure S10: iR-compensated polarization curves of HMFEOR over CF, Co(OH)F/CF, CoO/CF, CoO@ZIF-67/CF, and Co-CoO@C/CF; Figure S11: Polarization curves of CoO-Co@C/CF at a scan rate of 10 mV·s^-1^ in 1.0 M of KOH with 10 mM various biomass substrates; Figures S12–S16: Measurements of HMF, FDCA, DFF, HMFCA, and FFCA by HPLC and corresponding standard curve; Figure S17: The photographs of H-type cell (the right side: HMFEOR cell and the left side: HER cell) before (a) and after (b) HMFEOR; Figure S18: I–t curve for CoO-Co@C/CF at various constant potential in 1.0 M KOH with 10 mM HMF by passing the charge of 58 C; Figure S19: iR-compensated polarization curves of HER over CoO-Co@C/CF, CF, Co(OH)F/CF, CoO/CF, and CoO@ZIF-67/CF; Figure S20: SEM images of CoO-Co@C/CF after HMFEOR (a,b) and HER (c,d) stability test; Figure S21: XRD patterns of CoO-Co@C/CF after stability test; Figure S22: The stability test of CoO-Co@C/CF||CoO-Co@C/CF system in 1.0 M KOH with 10 mM HMF at voltage of 1.45 V. (a) I–t curve of 6 cycles and (b) corresponding HMF conversion and FDCA yield. Table S1: Parameters obtained by fitting the Nyquist plots of CoO-Co@C/CF, CoO@C/CF and Co@C/CF measured in 1.0 M KOH with 10 mM HMF; Tables S2 and S3: Comparison of HMFEOR and HER performance for CoO-Co@C/CF with recently reported electrocatalysts; Table S4: Comparison of H_2_O-HMF paired electrolysis on CoO-Co@C/CF with recently reported electrocatalysts in alkaline electrolyte [50,51,52,53,54,55,56,57,58,59,60,61,62,63,64,65,66,67,68,69,70,71,72,73,74,75,76,77,78,79,80].

## Sample Availability

Samples of the compounds are available from the authors.
