# Supplementary material for: CoO–Co Heterojunction Covered with Carbon Enables Highly Efficient Integration of Hydrogen Evolution and 5-Hydroxymethylfurfural Oxidation
Source: Molecules. 2023 Mar 29;28(7):3040. doi: 10.3390/molecules28073040 (PMC10096219; doi:10.3390/molecules28073040)
Supplement: Supplementary file 1 [file molecules-28-03040-s001.zip › molecules-2297765-supplementary.pdf]

Supplementary Material

CoO-Co Heterojunction Covered with Carbon Enables Highly Efficient Integration of Hydrogen Evolution and 5-Hydroxymethylfurfural Oxidation

Lei Zhao, Shichao Du *, Rong Gong, Wanqi Jia, Zhimin Chen and Zhiyu Ren *

Key Laboratory of Functional Inorganic Material Chemistry (Ministry of Education of China), School of Chemistry and Materials Science, Heilongjiang University, Harbin 150080, P. R. China.
* Correspondence: zyren@hlju.edu.cn

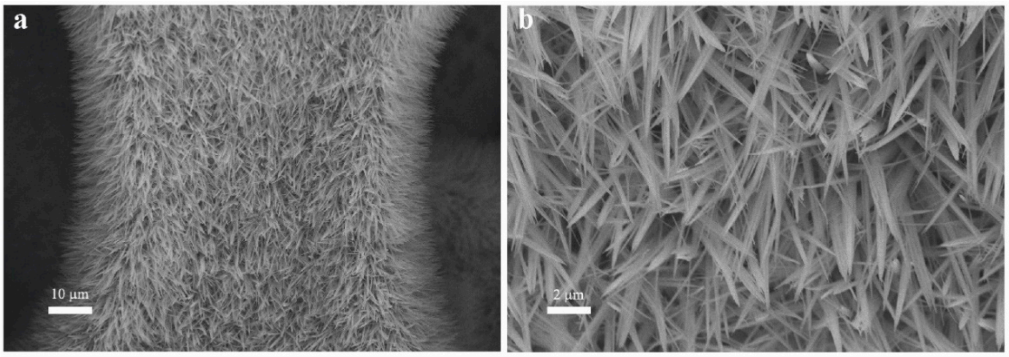

Figure S1. Typical SEM images of Co(OH)F/CF nanocones.

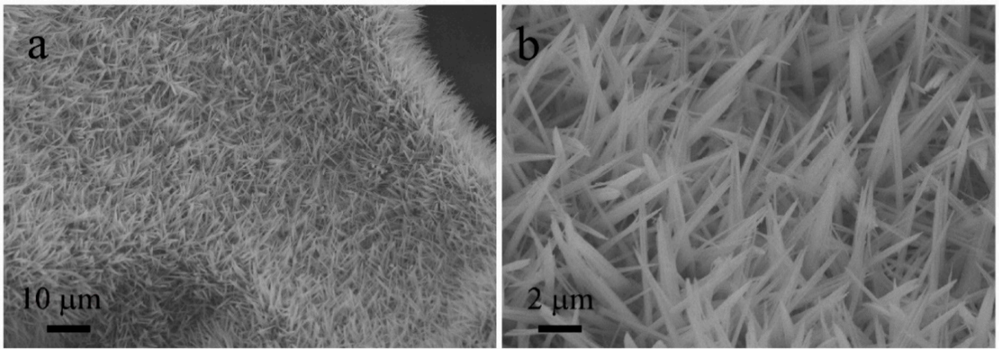

Figure S2. Typical SEM images of CoO/CF.

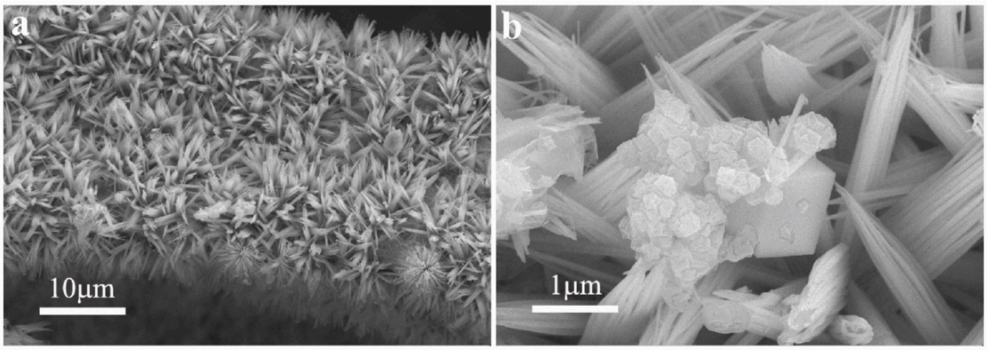

Figure S3. Typical SEM images of Co(OH)F@ZIF-67/CF.

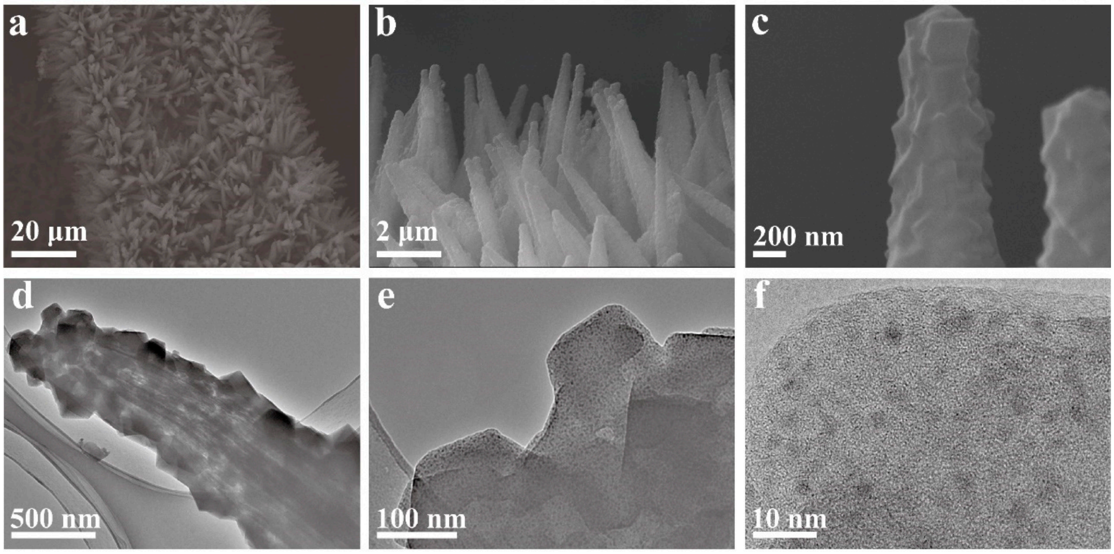

Figure S4. Typical SEM images (a-c), TEM images (d-e) and high-resolution TEM image of CoO@ZIF-67/CF.

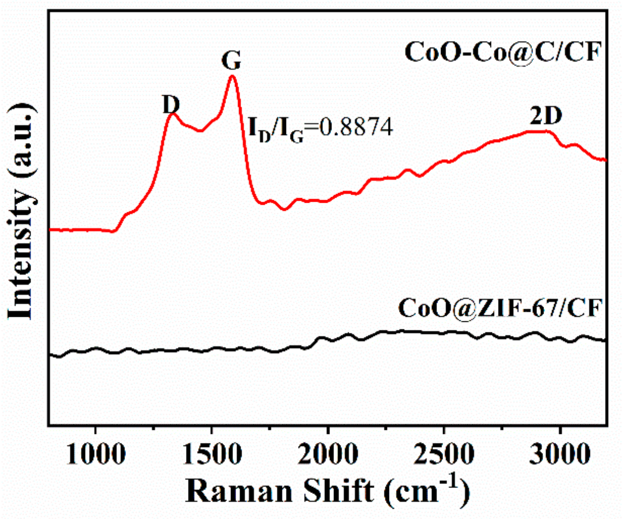

Figure S5. The Raman spectra of the CoO-Co@C/CF and CoO@ZIF-67/CF.

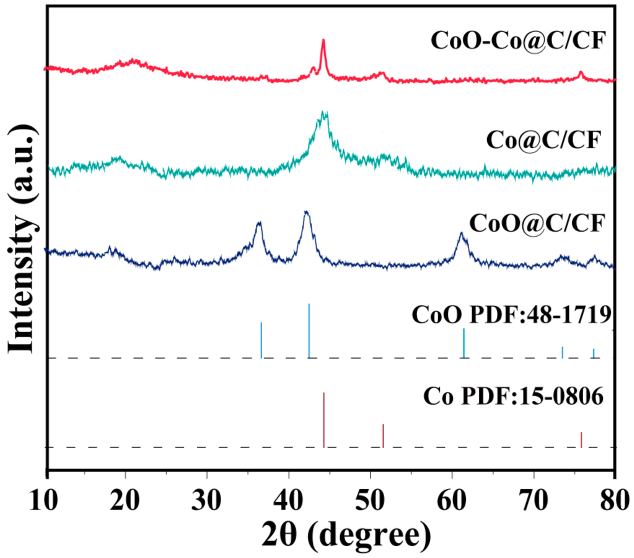

Figure S6. XRD patterns of CoO-Co@C/CF, CoO@C/CF and Co@C/CF.

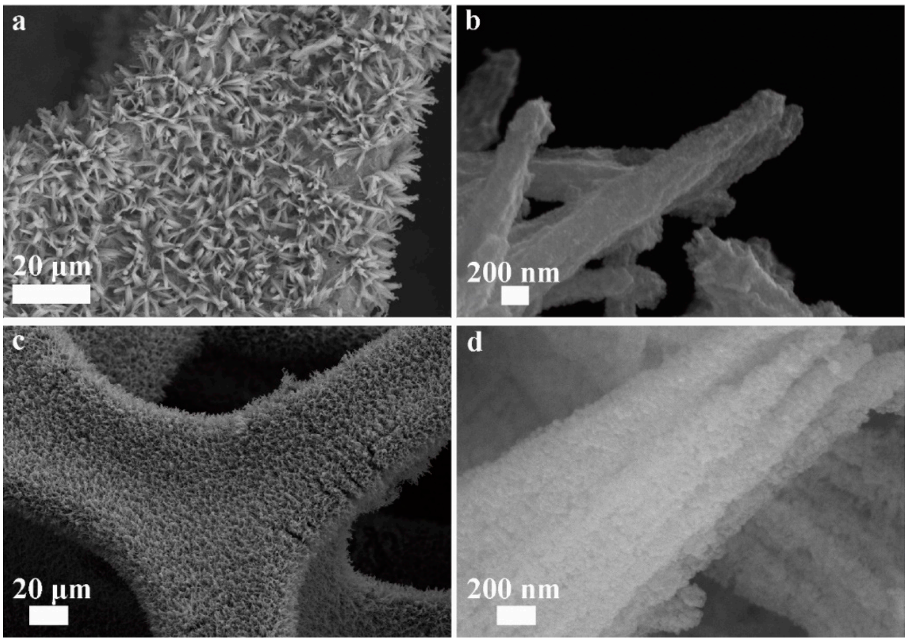

Figure S7. Typical SEM images of CoO@C/CF (a and b), Co@C/CF (c and d).

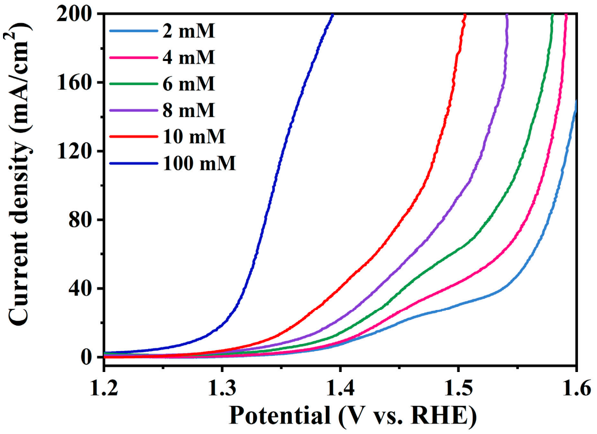

Figure S8. Polarization curves of HMFEOR over CoO-Co@C/CF in 1.0 M KOH with various concentrations of HMF (2, 4, 6, 8, 10 and 100 mM) at the scan rate of 5 mV·s^{-1}.

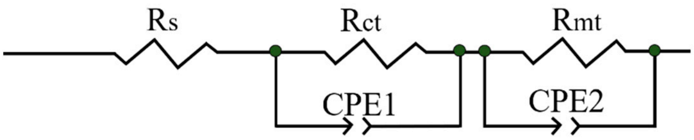

Figure S9. The equivalent circuit model of Nyquist plots.

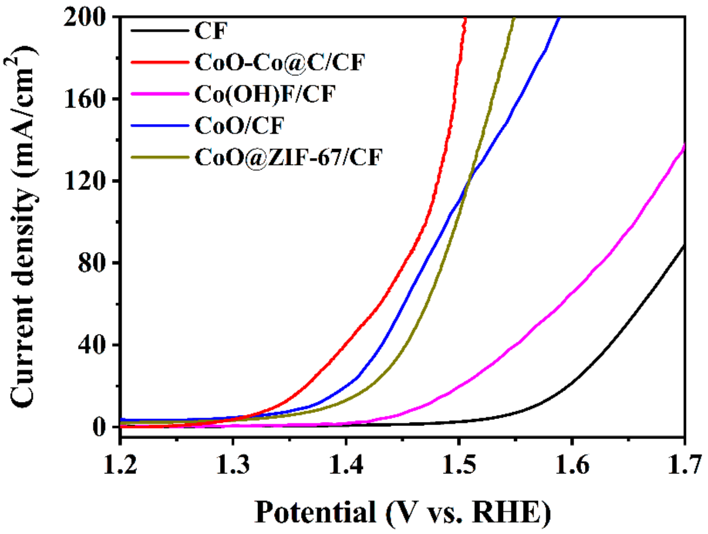

Figure S10. *iR*-compensated polarization curves of HMFEOR over CF, Co(OH)F/CF, CoO/CF, CoO@ZIF-67/CF, and Co-CoO@C/CF in 1.0 M KOH with 10 mM HMF at the scan rate of 5 mV·s^{-1}.

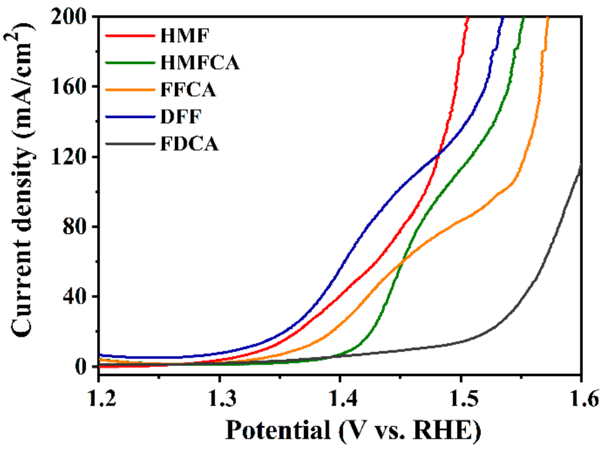

Figure S11. Polarization curves of CoO-Co@C/CF at a scan rate of 10 mV·s^{-1} in 1.0 M of KOH with 10 mM various biomass substrates (HMF, HMFCA, FFCA, DFF or FDCA).

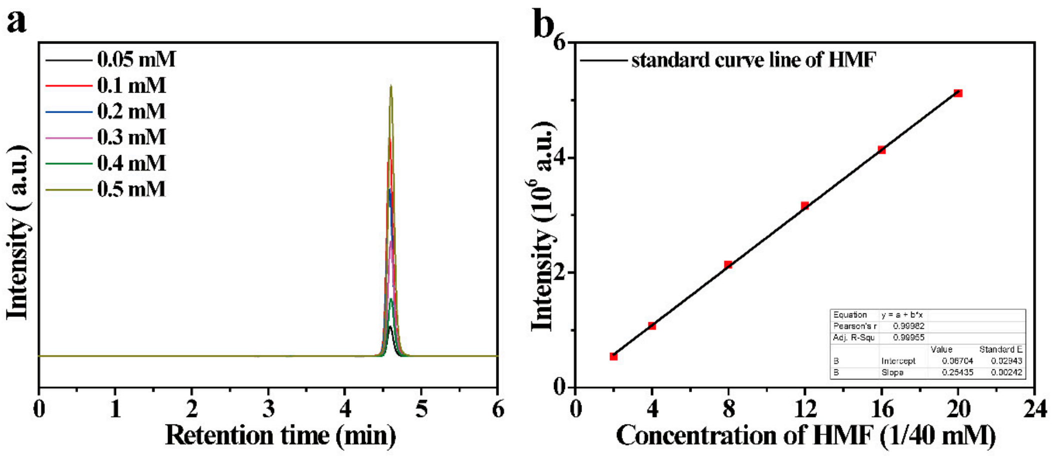

Figure S12. Measurements of HMF by HPLC and corresponding standard curve.

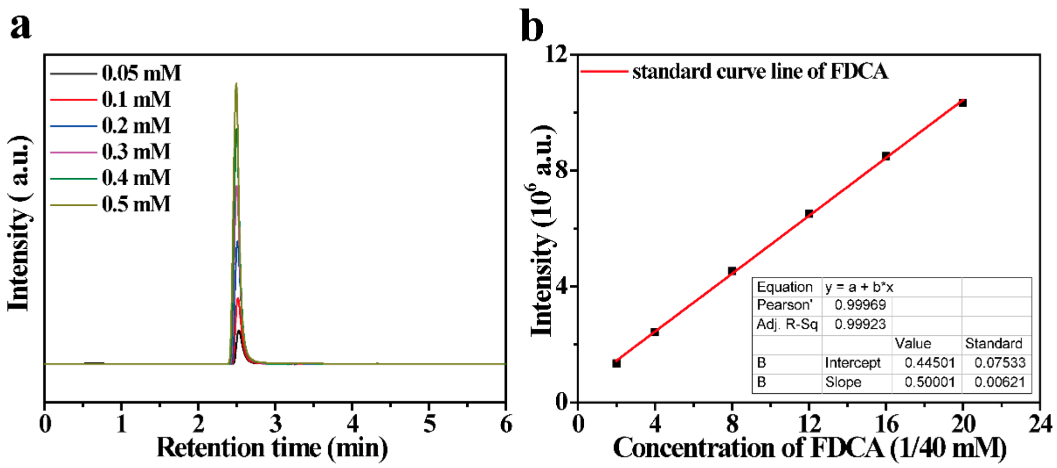

Figure S13. Measurements of FDCA by HPLC and corresponding standard curve.

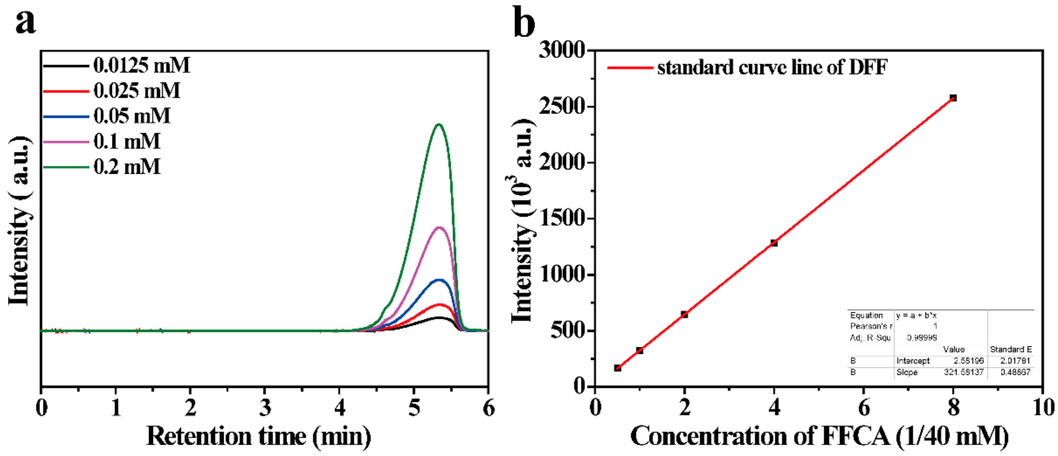

Figure S14. Measurements of DFF by HPLC and corresponding standard curve.

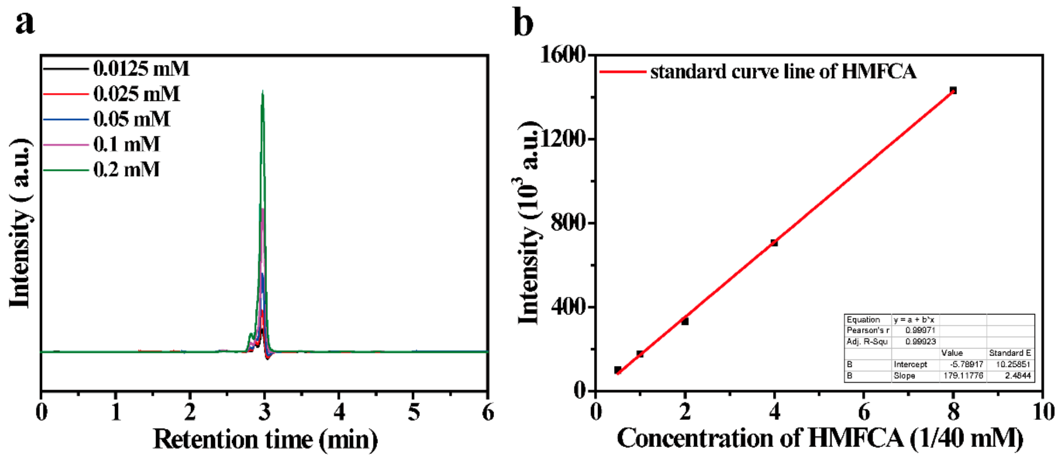

Figure S15. Measurements of HMFCA by HPLC and corresponding standard curve.

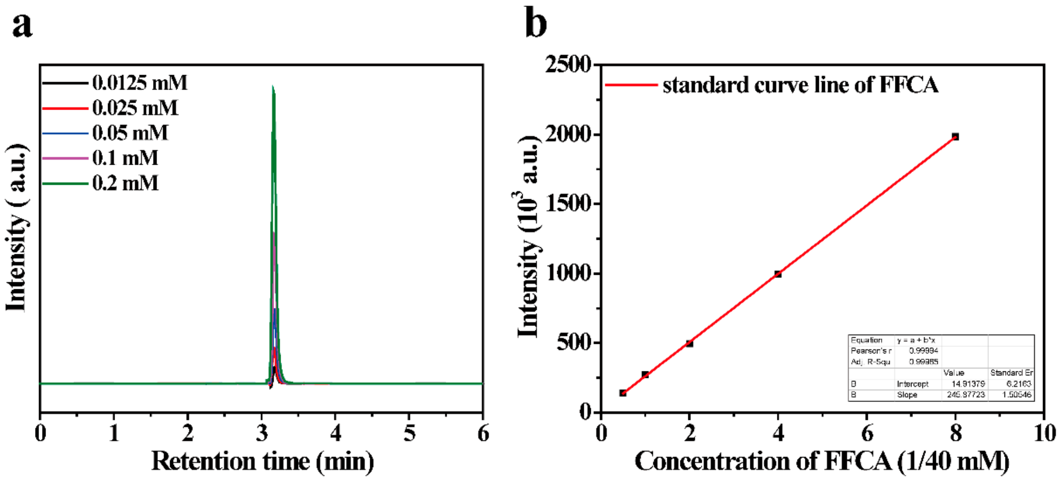

Figure S16. Measurements of FFCA by HPLC and corresponding standard curve.

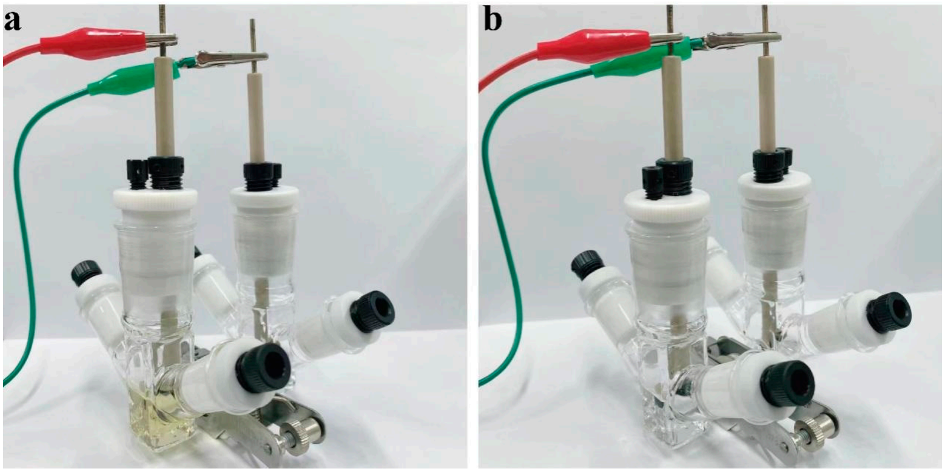

Figure S17. The photographs of H-type cell (the right side: HMFEOR cell and the left side: HER cell) before (a) and after (b) HMFEOR (Charge: 58 C, a potential of 1.37 V vs. RHE for 2.4 h).

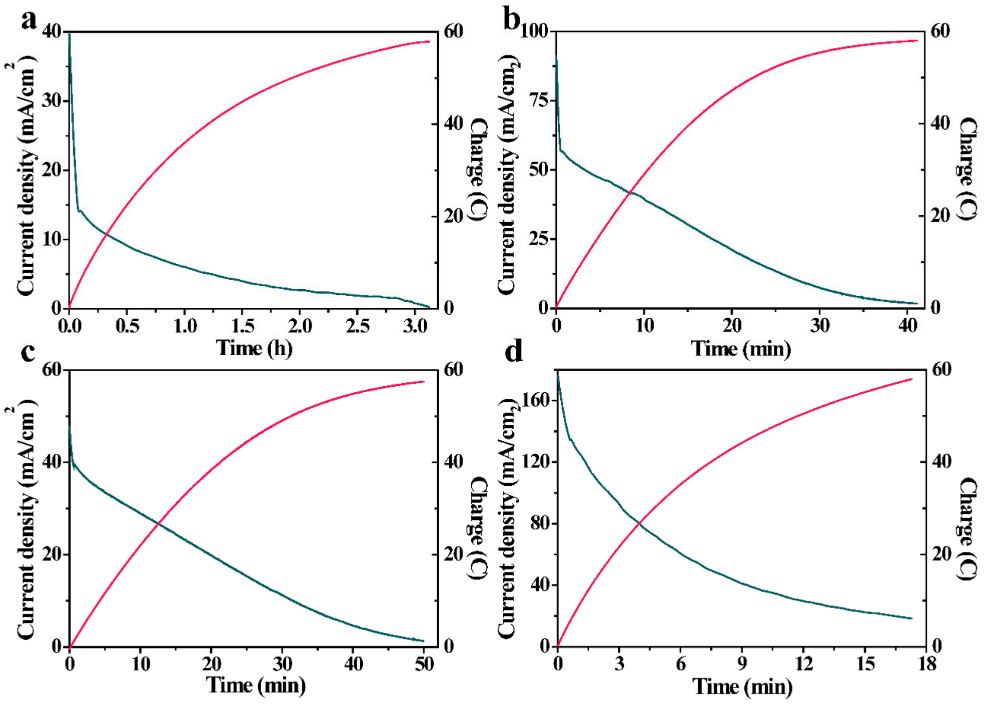

Figure S18. I-t curve for CoO-Co@C/CF at various constant potential in 1.0 M KOH with 10 mM HMF by passing the charge of 58 C, the constant potential is 1.34 V vs. RHE (a), 1.42 V vs. RHE (b), 1.47 V vs. RHE (c), 1.52 V vs. RHE (d), respectively.

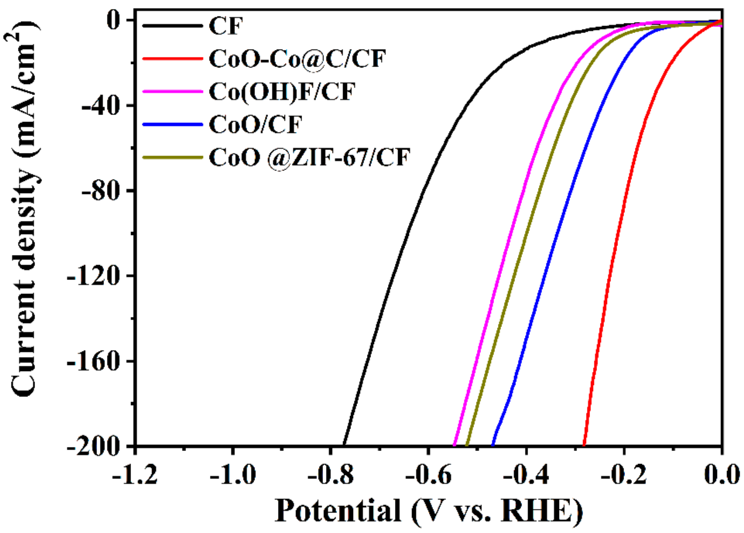

Figure S19. *iR*-compensated polarization curves of HER over CoO-Co@C/CF, CF, Co(OH)F/CF, CoO/CF, and CoO@ZIF-67/CF in 1.0 M KOH at a scan rate of 5 mV·s^{-1}.

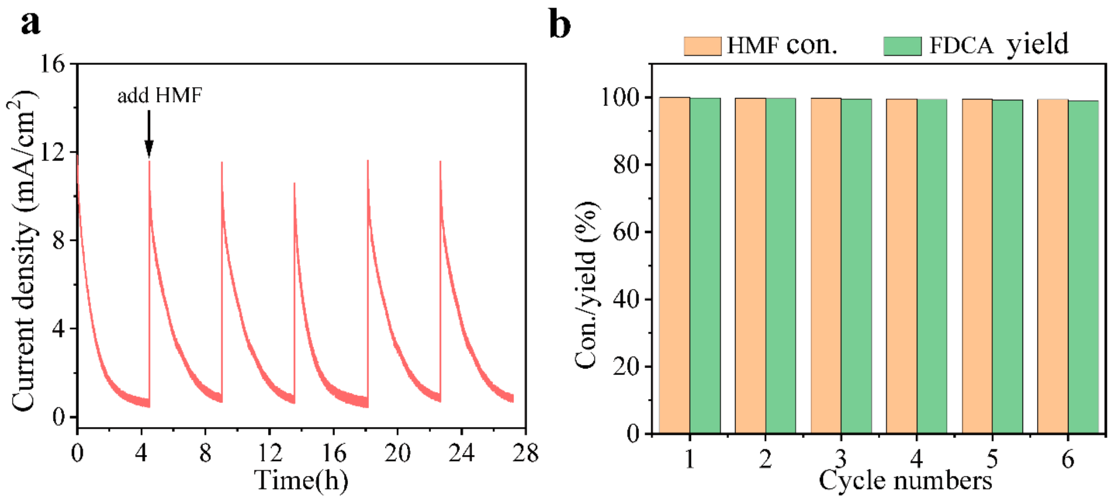

Figure S20. The stability test of CoO-Co@C/CF||CoO-Co@C/CF system in 1.0 M KOH with the intermittent addition of 10 mM HMF at voltage of 1.45 V, I-t curves of 6 cycles (a) and the corresponding HMF conversion and FDCA yield (b).

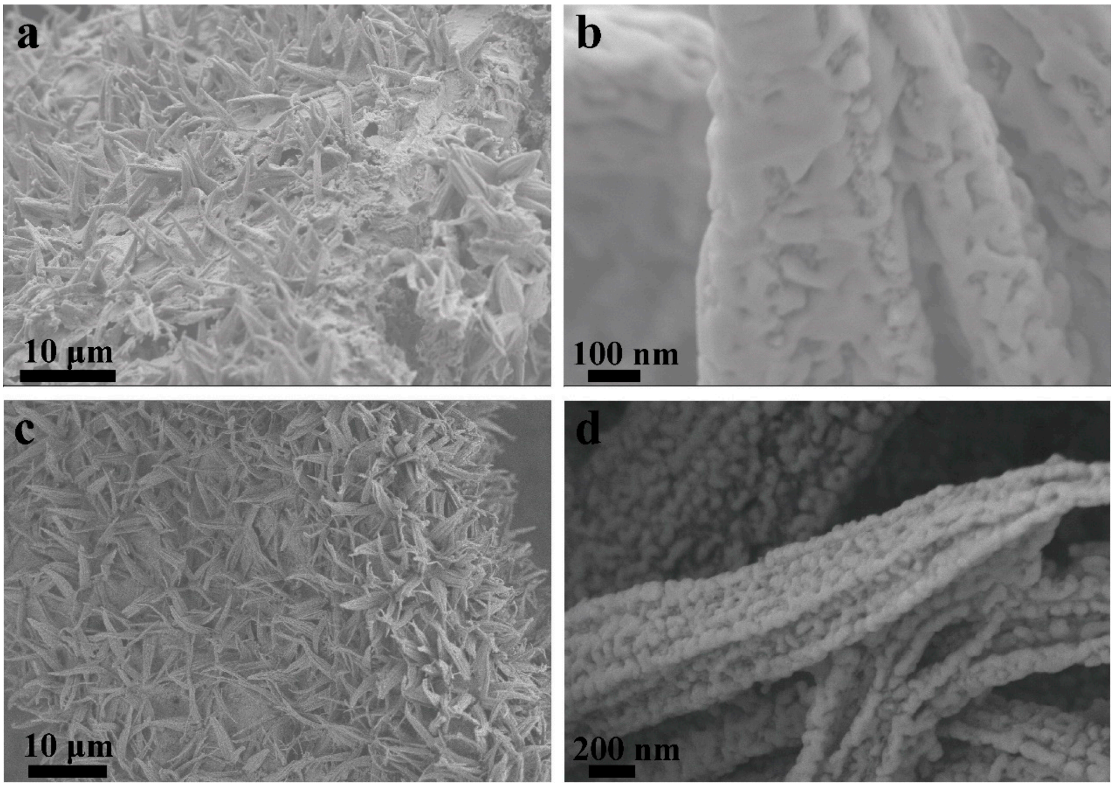

Figure S21. SEM images of CoO-Co@C/CF after HMFEOR (a-b) and HER (c-d).

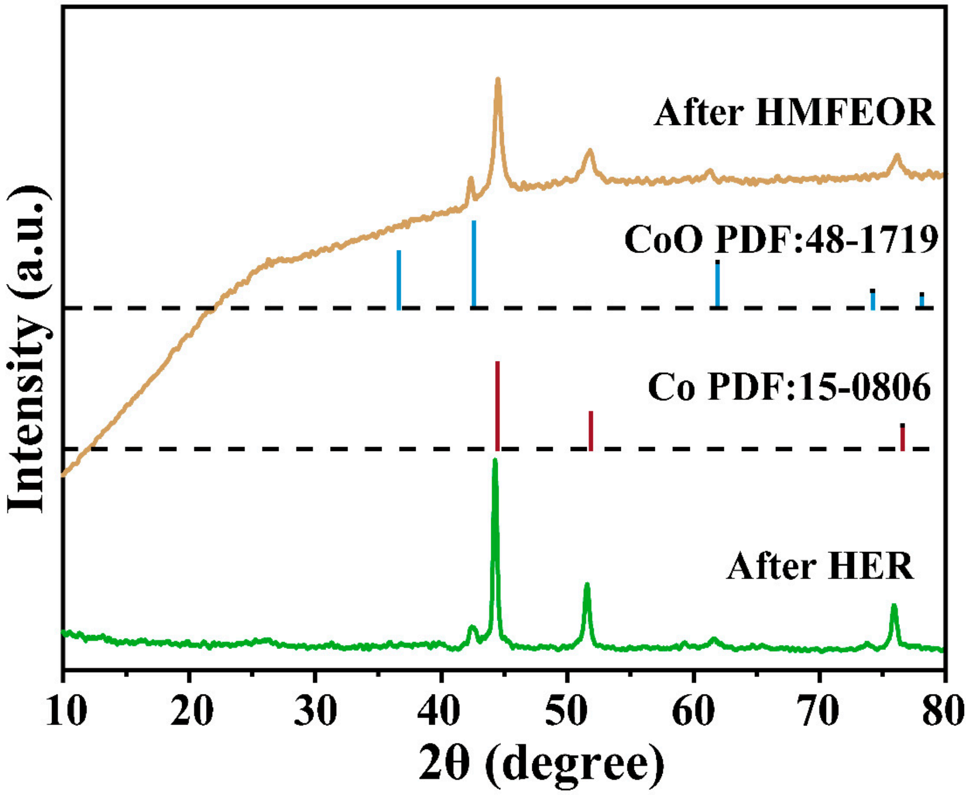

Figure S22. XRD patterns of CoO-Co@C/CF after HMFEOR and HER.

Table S1. Parameters obtained by fitting the Nyquist plots of CoO-Co@C/CF, CoO@C/CF and Co@C/CF measured in 1.0 M KOH with 10 mM HMF based on **Figure S9**.

Catalyst	R_{ct} (Ω)	
	HMFEOR (1.34 V vs. RHE)	HER (80 mV vs. RHE)
Co@C/CF	5.13	5.2
CoO@C/CF	39.7	38.3
Co-CoO@C/CF	58.1	26.4

Table S2. Comparison of HMFEOR performance for CoO-Co@C/CF with recently reported electrocatalysts.

**Catalysts**	**Oxidation potential (V)**	**Conversion (%)**	**Selectivity (%)**	**FE (%)**	**Reference**
$NiCo_2O_4$/NF	1.36 (onset)	99.6	90.8	80	[50]
$NiCo_2O_4$	1.50 (20 mA/cm^2)	90.8	90.8	87.5	[50]
CoNW/NF	1.311 (10 mA/cm^2)	100	96.82	/	[51]
$CuNi(OH)_2$	1.45 (4.6 mA/cm^2)	>99	93.3	93	[52]
$Ni(OH)_2$/NiOOH	1.34 (onset)	99.8	96.0	96.0	[53]
NiSe@NiOx	1.35 (onset)	99	98	98	[54]
Ni_3N@C	1.36 (20 mA/cm^2)	/	98	99	[55]
CoNiFe LDH	1.55 (20 mA/cm^2)	84.9	84.9	90	[21]
NiS_x/Ni_2P	1.346 (20 mA/cm^2)	98.5	98.5	95.1	[56]
Co_9S_8–Ni_3S_2@NSOC	1.330 (10 mA/cm^2)	99.5	98.8	98.6	[57]
om-Co3O4	1.45 (20 mA/cm^2)	>99.8	>99.8	>99.8	[58]
$NiCo_2O_4$-CFP	1.43 (50 mA/cm^2)	98.4	94.3	89.6	[59]
$Cu_{0.5}Co_{2.5}$O4	1.55 (20 mA/cm^2)	90	93	91	[60]
CoO@C/CF	1.37 (10 mA/cm^2)	90	84	82	This work
Co@C/CF	1.38 (10 mA/cm^2)	80	74	72	This work
CoO-Co@C/CF	1.34 (10 mA/cm^2)	100	99.4	99.4	This work

Table S3. Comparison of the HER performance for CoO-Co@C/CF with recently reported Co-based electrocatalysts in 1 M KOH electrolyte.

**Catalysts**	**Potential (mV) at 10 mA cm^{-2}**	**Reference**
CoOx@CN	232	[61]
CoO@Co/N-rGO	237	[62]
Co@NG	200	[62]
Co@CFNG@NF	212	[63]
Co@NC/CFP	178	[64]
Co@N-CS/N-HCP@CC	66	[65]
CoNi@NC	142	[66]
CoNi@NC-NCNTs	85	[67]
Co@NC/CNT	203	[68]
Co/CoO@NC@CC	152	[69]
$Cu_{0.5}Co_{2.5}O_4$	103	[60]
$CuCo_2O_4$	144	[60]
Co_3O_4	180	[60]
CuxS@NiCo-LDH	107	[24]
Co_3O_4/NF	105	[70]
$NiCo_2S_4$	345	[71]
CoNiBDC/CC	135	[72]
$Co(OH)_2$@P-NiCo-LDH	226	[73]
$NiCo_2O_4$/NiCoP	198	[74]
CoO@C/CF	190	This work
Co@C/CF	94	This work
CoO-Co@C/CF	69	This work

Table S4. Comparison of H_2O-HMF paired electrolysis on CoO-Co@C/CF with recently reported electrocatalysts in alkaline electrolyte.

Electrode Assembly	Anodic Reaction	Cell performance	Reference
Ni_3S_2/NF||Ni_3S_2/NF	HMF oxidation	1.58 V (50 mA/cm^2)<br>1.64 V (100 mA/cm^2)	[41]
Ni_2P/NF||Ni_2P/NF	HMF oxidation	1.5 V (50 mA/cm^2)<br>1.63 V (100 mA/cm^2)	[17]
NC@$CuCo_2N_x$/CF|| NC@$CuCo_2N_x$/CF	Benzyl Alcohol	1.56 V (10 mA/cm^2)	[75]
NiMoO-Ar/NF|| NiMoO-H_2/NF	Urea oxidation	1.48 V (50 mA/cm^2)<br>1.55 V (100 mA/cm^2)	[76]
NiSe/NF||CoP	Amine oxidation	1.44 V (10 mA/cm^2)	[77]
Co(OH)F/NF||CoP/NF	Urea oxidation	1.42 V (20 mA/cm^2)	[78]
CoNW/NF || CoNW/NF	HMF oxidation	1.504 V (10 mA/cm^2)	[51]
$Ni_{0.33}Co_{0.67}(OH)_2$/NF || $Ni_{0.33}Co_{0.67}(OH)_2$/NF	Methanol oxidation	1.65 V (50 mA/cm^2)<br>1.75 V (100 mA/cm^2)	[79]
Cu_xS@$Ni_{0.75}Co_{0.25}$OmHnll<br>Cu_xS@$Ni_{0.75}Co_{0.25}$OmHn	HMF oxidation	1.49 V (50 mA/cm^2)<br>1.58 V (100 mA/cm^2)	[24]
MoO_2-FeP@C || MoO_2-FeP@C	HMF oxidation	1.486 V (10 mA/cm^2)	[80]
CoO@C/CF	HMF oxidation	1.502 V (10 mA/cm^2)	This work
Co@C/CF	HMF oxidation	1.552 V (10 mA/cm^2)	This work
CoO-Co@C/CF	HMF oxidation	1.448 V (10 mA/cm^2)	This work

Reference

17. You, B.; Jiang, N.; Liu, X.; Sun, Y. Simultaneous H_2 Generation and Biomass Upgrading in Water by an Efficient Noble-Metal-Free Bifunctional Electrocatalyst. *Angew. Chem. Int. Ed.* **2016**, *55*, 9913-9917, doi:10.1002/anie.201603798.

21. Zhang, M.; Liu, Y.; Liu, B.; Chen, Z.; Xu, H.; Yan, K. Trimetallic NiCoFe-Layered Double Hydroxides Nanosheets Efficient for Oxygen Evolution and Highly Selective Oxidation of Biomass-Derived 5-Hydroxymethylfurfural. *ACS Catal.* **2020**, *10*, 5179-5189, doi:10.1021/acscatal.0c00007.

24. Deng, X.; Kang, X.; Li, M.; Xiang, K.; Wang, C.; Guo, Z.; Zhang, J.; Fu, X.-Z.; Luo, J.-L. Coupling efficient biomass upgrading with H_2 production via bifunctional Cu_xS@NiCo-LDH core–shell nanoarray electrocatalysts. *J. Mater. Chem. A* **2020**, *8*, 1138-1146, doi:10.1039/C9TA06917H.

41. You, B.; Liu, X.; Jiang, N.; Sun, Y.J. A General Strategy for Decoupled Hydrogen Production from Water Splitting by Integrating Oxidative Biomass Valorization. *J. Am. Chem. Soc.* **2016**, *138*, 13639-13646, doi:10.1021/jacs.6b07127.

50. Kang, M.J.; Park, H.; Jegal, J.; Hwang, S.Y.; Kang, Y.S.; Cha, H.G. Electrocatalysis of 5-hydroxymethylfurfural at cobalt based

spinel catalysts with filamentous nanoarchitecture in alkaline media. *Appl. Catal. B: Environ.* **2019**, *242*, 85-91, doi: 10.1016/j.apcatb.2018.09.087.

51. Zhou, Z.; Chen, C.; Gao, M.; Xia, B.; Zhang, J. In situ anchoring of a Co_3O_4 nanowire on nickel foam: an outstanding bifunctional catalyst for energy-saving simultaneous reactions. *Green Chem.* **2019**, *21*, 6699-6706, doi:10.1039/C9GC02880C.
52. Chen, H.; Wang, J.T.; Yao, Y.; Zhang, Z.H.; Yang, Z.Z.; Li, J.; Chen, K.Q.; Lu, X.Y.; Ouyang, P.K.; Fu, J. Cu-Ni Bimetallic Hydroxide Catalyst for Efficient Electrochemical Conversion of 5-Hydroxymethylfurfural to 2,5-Furandicarboxylic Acid. *Chemelectrochem* **2019**, *6*, 5797-5801, doi:10.1002/celc.201901366.
53. Taitt, B.J.; Nam, D.-H.; Choi, K.-S. A Comparative Study of Nickel, Cobalt, and Iron Oxyhydroxide Anodes for the Electrochemical Oxidation of 5-Hydroxymethylfurfural to 2,5-Furandicarboxylic Acid. *ACS Catal.* **2019**, *9*, 660-670, doi:10.1021/acscatal.8b04003.
54. Gao, L.; Liu, Z.; Ma, J.; Zhong, L.; Song, Z.; Xu, J.; Gan, S.; Han, D.; Niu, L. NiSe@NiO_x core-shell nanowires as a non-precious electrocatalyst for upgrading 5-hydroxymethylfurfural into 2,5-furandicarboxylic acid. *Appl. Catal. B: Environ.* **2020**, *261*, doi:10.1016/j.apcatb.2019.118235.
55. Zhang, N.; Zou, Y.; Tao, L.; Chen, W.; Zhou, L.; Liu, Z.; Zhou, B.; Huang, G.; Lin, H.; Wang, S. Electrochemical Oxidation of 5-Hydroxymethylfurfural on Nickel Nitride/Carbon Nanosheets: Reaction Pathway Determined by In Situ Sum Frequency Generation Vibrational Spectroscopy. *Angew. Chem. Int. Ed.* **2019**, *58*, 15895-15903, doi: 10.1002/anie.201908722.
56. Zhang, B.; Fu, H.; Mu, T. Hierarchical NiS_x/Ni_2P nanotube arrays with abundant interfaces for efficient electrocatalytic oxidation of 5-hydroxymethylfurfural. *Green Chem.* **2022**, *24*, 877-884, doi:10.1039/D1GC04206H.
57. Zhang, Y.; Xue, Z.; Zhao, X.; Zhang, B.; Mu, T. Controllable and facile preparation of Co_9S_8–Ni_3S_2 heterostructures embedded with N,S,O-tri-doped carbon for electrocatalytic oxidation of 5-hydroxymethylfurfural. *Green Chem.* **2022**, *24*, 1721-1731, doi:10.1039/D1GC04499K.
58. Wang, C.; Bongard, H.-J.; Yu, M.; Schüth, F. Highly Ordered Mesoporous Co_3O_4 Electrocatalyst for Efficient, Selective, and Stable Oxidation of 5-Hydroxymethylfurfural to 2,5-Furandicarboxylic Acid. *Chemsuschem* **2021**, *14*, 5199-5206, doi: 10.1002/cssc.202002762.
59. Zhong, Y.; Ren, R.-Q.; Qin, L.; Wang, J.-B.; Peng, Y.-Y.; Li, Q.; Fan, Y.-M. Electrodeposition of hybrid nanosheet-structured $NiCo_2O_4$ on carbon fiber paper as a non-noble electrocatalyst for efficient electrooxidation of 5-hydroxymethylfurfural to 2,5-furandicarboxylic acid. *New J. Chem.* **2021**, *45*, 11213-11221, doi:10.1039/D1NJ01489G.
60. Tao, Y.; Fan, S.; Li, X.; Yang, J.; Wang, J.; Tadé, M.O.; Liu, S. $Cu_xCo_{3-x}O_4$ Spinel Nanofibers for Selective Oxidation of 5-Hydroxymethylfurfural into Fuel Additives. *ACS Appl. Nano Mater.* **2022**, *5*, 16564-16572, doi:10.1021/acsanm.2c03656.
61. Jin, H.; Wang, J.; Su, D.; Wei, Z.; Pang, Z.; Wang, Y. In situ Cobalt–Cobalt Oxide/N-Doped Carbon Hybrids As Superior Bifunctional Electrocatalysts for Hydrogen and Oxygen Evolution. *J. Am. Chem. Soc.* **2015**, *137*, 2688-2694, doi:10.1021/ja5127165.
62. Liu, X.X.; Zang, J.B.; Chen, L.; Chen, L.B.; Chen, X.; Wu, P.; Zhou, S.Y.; Wang, Y.H. A microwave-assisted synthesis of CoO@Co core–shell structures coupled with N-doped reduced graphene oxide used as a superior multi-functional electrocatalyst for hydrogen evolution, oxygen reduction and oxygen evolution reactions. *J. Mater. Chem. A* **2017**, *5*, 5865-5872, doi:10.1039/C6TA10591B.
63. Pei, Z.; Tang, Z.; Liu, Z.; Huang, Y.; Wang, Y.; Li, H.; Xue, Q.; Zhu, M.; Tang, D.; Zhi, C. Construction of a hierarchical 3D Co/N-carbon electrocatalyst for efficient oxygen reduction and overall water splitting. *J. Mater. Chem. A* **2018**, *6*, 489-497, doi:10.1039/C7TA09254G.
64. Wang, Z.-L.; Hao, X.-F.; Jiang, Z.; Sun, X.-P.; Xu, D.; Wang, J.; Zhong, H.-X.; Meng, F.-L.; Zhang, X.-B. C and N Hybrid Coordination Derived Co–C–N Complex as a Highly Efficient Electrocatalyst for Hydrogen Evolution Reaction. *J. Am. Chem. Soc.* **2015**, *137*, 15070-15073, doi:10.1021/jacs.5b09021.
65. Chen, Z.; Ha, Y.; Jia, H.; Yan, X.; Chen, M.; Liu, M.; Wu, R. Water Splitting: Oriented Transformation of Co-LDH into 2D/3D ZIF-67 to Achieve Co–N–C Hybrids for Efficient Overall Water Splitting. *Adv. Energ. Mater.* **2019**, *9*, 1970066, doi:10.1002/aenm.201970066.
66. Deng, J.; Ren, P.; Deng, D.; Bao, X. Enhanced Electron Penetration through an Ultrathin Graphene Layer for Highly Efficient Catalysis of the Hydrogen Evolution Reaction. *Angew. Chem. Int. Ed.* **2015**, *54*, 2100-2104, doi: 10.1002/anie.201409524.
67. Li, R.; Li, X.; Liu, C.; Ye, M.; Yang, Q.; Liu, Z.; Xie, L.; Yang, G. Enhanced electron transport through a nanoforest-like structure of CoNi nanoalloy@nitrogen-doped carbon nanotubes for highly efficient catalysis of overall water splitting. *Appl. Surf. Sci.* **2020**, *517*, 145841, doi: 10.1016/j.apsusc.2020.145841.
68. Yang, F.; Zhao, P.; Hua, X.; Luo, W.; Cheng, G.; Xing, W.; Chen, S. A cobalt-based hybrid electrocatalyst derived from a carbon nanotube inserted metal–organic framework for efficient water-splitting. *J. Mater. Chem. A* **2016**, *4*, 16057-16063, doi:10.1039/C6TA05829A.
69. Dai, K.; Zhang, N.; Zhang, L.; Yin, L.; Zhao, Y.; Zhang, B. Self-supported Co/CoO anchored on N-doped carbon composite as bifunctional electrocatalyst for efficient overall water splitting. *Chem. Eng. J.* **2021**, *414*, 128804, doi:10.1016/j.cej.2021.128804.
70. Feng, Y.; Guo, H.; Smith, R.L.; Qi, X. Electrocatalytic oxidation of 5-hydroxymethylfurfural to 2,5-furandicarboxylic acid via metal-organic framework-structured hierarchical Co_3O_4 nanoplate arrays. *J. Colloid Interf. Sci.* **2023**, *632*, 87-94, doi:10.1016/j.jcis.2022.11.068.
71. Aftab, U.; Tahira, A.; Mazzaro, R.; Morandi, V.; Ishaq Abro, M.; Baloch, M.M.; Yu, C.; Ibupoto, Z.H. Nickel–cobalt bimetallic sulfide $NiCo_2S_4$ nanostructures for a robust hydrogen evolution reaction in acidic media. *RSC Adv.* **2020**, *10*, 22196-22203, doi:10.1039/D0RA03191G.
72. Bai, X.-J.; Chen, H.; Li, Y.-N.; Shao, L.; Ma, J.-C.; Li, L.-L.; Chen, J.-Y.; Wang, T.-Q.; Zhang, X.-M.; Zhang, L.-Y.; et al. CoNi-based metal–organic framework nanoarrays supported on carbon cloth as bifunctional electrocatalysts for efficient water-splitting.

New J. Chem. **2020**, *44*, 1694-1698, doi:10.1039/C9NJ06204A.
73. Song, N.; Hong, S.; Xiao, M.; Zuo, Y.; Jiang, E.; Li, C.; Dong, H. Fabrication of Co(Ni)-P surface bonding states on core–shell $Co(OH)_2$@P-NiCo-LDH towards electrocatalytic hydrogen evolution reaction. *J. Colloid Interf. Sci.* **2021**, *582*, 535-542, doi:10.1016/j.jcis.2020.08.086.
74. Jin, W.; Chen, J.; Wu, H.; Zang, N.; Li, Q.; Cai, W.; Wu, Z. Interface engineering of oxygen-vacancy-rich $NiCo_2O_4$/NiCoP heterostructure as an efficient bifunctional electrocatalyst for overall water splitting. *Catal. Sci. Tech.* **2020**, *10*, 5559-5565, doi:10.1039/D0CY01115K.
75. Zheng, J.; Chen, X.; Zhong, X.; Li, S.; Liu, T.; Zhuang, G.; Li, X.; Deng, S.; Mei, D.; Wang, J.-G. Hierarchical Porous NC@CuCo Nitride Nanosheet Networks: Highly Efficient Bifunctional Electrocatalyst for Overall Water Splitting and Selective Electrooxidation of Benzyl Alcohol. *Adv. Func. Mater.* **2017**, *27*, 1704169, doi:10.1002/adfm.201704169.
76. Yu, Z.-Y.; Lang, C.-C.; Gao, M.-R.; Chen, Y.; Fu, Q.-Q.; Duan, Y.; Yu, S.-H. Ni–Mo–O nanorod-derived composite catalysts for efficient alkaline water-to-hydrogen conversion via urea electrolysis. *Energ. Environ. Sci.* **2018**, *11*, 1890-1897, doi:10.1039/C8EE00521D.
77. Huang, Y.; Chong, X.; Liu, C.; Liang, Y.; Zhang, B. Boosting Hydrogen Production by Anodic Oxidation of Primary Amines over a NiSe Nanorod Electrode. *Angew. Chem. Int. Ed.* **2018**, *57*, 13163-13166, doi:10.1002/anie.201807717.
78. Song, M.; Zhang, Z.; Li, Q.; Jin, W.; Wu, Z.; Fu, G.; Liu, X. Ni-foam supported Co(OH)F and Co–P nanoarrays for energy-efficient hydrogen production via urea electrolysis. *J. Mater. Chem. A* **2019**, *7*, 3697-3703, doi:10.1039/C8TA10985K.
79. Li, M.; Deng, X.; Xiang, K.; Liang, Y.; Zhao, B.; Hao, J.; Luo, J.-L.; Fu, X.-Z. Value-Added Formate Production from Selective Methanol Oxidation as Anodic Reaction to Enhance Electrochemical Hydrogen Cogeneration. *Chemsuschem* **2020**, *13*, 914-921, doi: 10.1002/cssc.201902921.
80. Yang, G.; Jiao, Y.; Yan, H.; Xie, Y.; Wu, A.; Dong, X.; Guo, D.; Tian, C.; Fu, H. Interfacial Engineering of MoO_2-FeP Heterojunction for Highly Efficient Hydrogen Evolution Coupled with Biomass Electrooxidation. *Adv Mater* **2020**, *32*, e2000455, doi:10.1002/adma.202000455.
